# 

**Published:** 1894-07

**Authors:** 


### CONTENTS:

### SELECTIONS.

Article I.—Immediate Root Filling. By Dr. Edmond Noyes. - 97
" II.—The Cracking of Teeth in Soldering. L. P. Heskell, D. D. S. - - - - - - - - 106
" III.—To Properly Articulate a Set of Teeth. - - 107
" IV.—The Semi-Centennial of the Discovery of Anæsthesia. 110
" V.—The Treatment of Pericementitis. By C. N. Johnson. 111
" VI.—Queer Facts About Teeth. - - - - - 116
" VII.—Gum Lancing in Difficult Primary Dentition. By Dr. E. C. Kirk, D. D. S. - - - - - 120
" VIII.—Tin, and Its Combinations with Gold and Amalgam. By S. B. Palmer, M. D. S. - - - - - 130

### EDITORIAL, Etc.

Governor Brown turned down the Dentists' Bill. - - - - 138
Lauder Brunton on Headaches. - - - - - - - 138
Opinion delivered by President Judge Hanna, Orphans' Court, Philadelphia, in the Waesch's Estate Case, - - - - 138

### MONTHLY SUMMARY.

Death after Taking Gas. - - - - - - - 138

Carborundum. - - - - - - - - 140

The Fourth Molar. - - - - - - - - 140

How to Polish Porcelain Teeth. - - - - - - 141

Soldering Aluminum. - - - - - - - 141

Anæsthesia. - - - - - - - - - 142

A Dental Revolt. - - - - - - - - 142

Oxiphosphate. - - - - - - - - 143

The Earliest Hospital on Record. - - - - - - 143

Capping Dental Pulps. - - - - - - - 144

American Dentists. - - - - - - - - 144

